# Lessons Learned: A Case for Reforming Siloed, Referral-Based Oncologic Palliative Care for High-Risk Opioid Patients

**DOI:** 10.1177/26892820251376975

**Published:** 2025-09-09

**Authors:** Benjamin Bailey, Georgetta Bundley, Hannah Bromberg

**Affiliations:** Department of Supportive Care Medicine, Moffitt Cancer Center, Tampa, Florida, USA.

**Keywords:** care delivery, multidisciplinary, opioid use disorder, referral-based, substance use disorder

## Abstract

The breadth and complexity of knowledge and services required for the delivery of medical care has resulted in team member specialization and subspecialization to meet the needs of our patients. As a collateral effect, medical care has become increasingly fragmented and insular with various barriers to provider collaboration. Yet, the more medically or psychosocially complex the patient, the greater the number of involved parties and the greater the need for interdisciplinary cooperation. The cited oncologic case highlights this issue within palliative care as it relates to the care of patients at elevated risk for opioid pain management. The patient’s clinical course and care barriers will be highlighted with discussion of potential areas for growth and reform, including earlier collaboration and case review, intensified case management, and early connection to advanced nonopioid pain interventions.

## Introduction

Opioids remain a central tool for the management of pain for palliative care providers. However, even in the setting of palliative care, there is growing recognition that prognosis and risk are not uniform and that care is needed to ensure the benefits of opioids are balanced against their risks.^1^ Opioid risk assessment remains an area of interest and research, and there is a lack of clarity on the best approach in the oncologic population.^2^ While the prevalence of opioid use disorder (OUD) in the United States has been estimated at 2.0%, estimates of OUD rates in cancer patients have been limited by methodological heterogeneity.^3,4^ However, broader behavioral assessments have shown risky behavior such as opioid self-titration or use with other illicit substances to be common, occurring in roughly one in five patients in one oncologic palliative care cohort.^5^ Importantly, palliative care providers may vary in their comfort navigating opioid prescribing in patients engaging in hazardous opioid use.^6^ The following oncologic case highlights potential limitations of a referral-based model for the assessment and management of hazardous opioid use.

## Case Description

Mr. G is a middle-aged man diagnosed with stage IV cancer complicated by pathological compression fractures of the spine. Five months after establishing care at his comprehensive cancer center, palliative care was consulted for assistance with pain management. Contact was made during a brief inpatient admission for uncontrolled pain and followed by close outpatient follow-up. During the first clinic visit, Mr. G reported ongoing issues with 8/10 pain of an electric-like quality he reported feeling “in his bones.” He reported his back pain to be the worst but reported some relief with ketorolac while inpatient. Accordingly, nonsteroidal therapy with celecoxib was initiated, and Mr. G was continued on his prior immediate-release morphine, extended-relief morphine, and gabapentin for pain control. Due to mood and anxiety complaints, he was also referred to psychology and psychiatry by his palliative care provider.

### Substance use discovered

A few days after the initial palliative care visit, however, routine urine drug testing resulted confirming via mass spectrometry the presence of fentanyl and methamphetamine in addition to his prescribed morphine. This resulted in the patient being brought back to the clinic the following week to discuss these findings. Risks of continued use were discussed, and more frequent monitoring and patient visits were arranged.

### Consultations & initial referral for SUD treatment

About 2 weeks after his initial outpatient palliative care visit, Mr. G met with psychology. The assessment detailed a long history of struggles with mental health and substance use dating back to his teenage years. Virtual follow-up visits were scheduled for support. Four weeks after establishing with palliative care, he met with psychiatry for consultation. During the visit, the patient confirmed a desire to stop methamphetamine and fentanyl use but expressed feeling unable to do so on his own, citing his pain and fear of it worsening. He had engaged with substance use disorder (SUD) treatment in the past with benefit but was skeptical of the benefits of starting buprenorphine. Due to the severity of his pain, his polysubstance use, his mental health comorbidities, and his limited social support, residential treatment was recommended and accepted. Social work was consulted in the clinic to facilitate connection to treatment prior to the patient’s departure. Regrettably, this proved to be a complicated request, as the patient was without any insurance and lacked personal funds to pay for private treatment. Social work called facilities and collected a short list of options for the patient to consider for treatment. Meanwhile, psychiatry consulted with the treating palliative care provider, who agreed to help coordinate pain management at the treating facility. However, the palliative care provider also expressed to the patient that it was no longer felt safe to prescribe opioids without him receiving substance abuse treatment. The patient left clinic with his substance use resource list, a prescription for intranasal naloxone, and follow-up appointments for psychiatry and palliative care.

### Follow-up and care course

Unfortunately, Mr. G did not admit to a residential treatment program. He also did not return for his follow-up psychology, psychiatry, or palliative care visits. His cancer care over the coming months would be fraught with poor-quality PET scans due to protocol nonadherence and missed or rescheduled appointments. It would be more than 4 months before he would return to mental health, reaching out to psychiatry again for referrals for substance abuse treatment. He was able to present for a second psychiatric appointment at which he expressed a continued struggle with pain control with daily use of stimulants (methamphetamine or cocaine) and fentanyl. He remained open to treatment and requested substance use treatment resources again. He was encouraged to reconsider buprenorphine maintenance while he worked on connecting to community treatment programs. He was reluctant based on prior experience but did accept a script for sublingual buprenorphine/naloxone and was scheduled for a two-week follow-up. Again, sadly, he did not present for residential treatment. Per prescription monitoring reports, he only filled part of his script for his low-dose buprenorphine induction. He did not show up for his follow-up psychiatry appointment and was eventually lost to all follow-up.

## Discussion

While the risks of prescription opioids are not uniform and hazardous behavior can vary, Mr. G’s case highlights one high-risk patient archetype: the patient with active OUD who requires pain management.^1,7^ In retrospect, there are several areas in which care may have been enhanced through a different approach. For simplicity, these have been reduced to three areas:

1) Early collaboration and case review.

Typical referral-based models can offer the primary provider feedback and assistance in the management of a patient. However, scheduling availability and additional office visits may add an extra layer of burden for the patient and delay collaboration. Opportunities for earlier case review may come from reducing consultation wait times or may come for alternative models for collaboration and review.

While there are different administrative means for reducing appointment wait times for specialists, there usually remains the problem of working with a finite resource. For SUDs, it is important to consider resources across multiple disciplines. Leveraging the expertise of other trained professionals, such as licensed counselors or clinical social workers with SUD experience, may allow for improved access and broader skill sets.

Regarding alternative models for co-management, a collaborative care-inspired model of partnership with an addiction psychiatrist and addiction counselor has been utilized in the palliative care setting to assist with co-management without necessarily requiring formal psychiatric consultation for all cases.^8^ The collaborative care model emphasizes systematic assessments, symptom tracking, and panel review with a specialist with more selective escalation to full consultation/evaluations. It does, however, rely on an additional care manager, such as a counselor, nurse, or social worker trained to assess and support with the target symptoms/behaviors and collaborate with the primary provider and medical specialist.^9^

Furthermore, at facilities with sufficient in-house specialty services, a tumor-board-style approach may be an alternative or supplement to a typical outpatient referral.^10^ As not all palliative care providers will have experience assessing and managing SUDs, early collaboration with an experienced provider may help with reducing barriers to the utilization of supportive medication and aid in the implementation of harm reduction practices.^7,8,11^

In the case of our patient, his SUD treatment was complicated by significant social barriers and was not easily amenable to on-the-spot planning. Also, all consultant recommendations regarding treatment plan alterations were restricted to independent evaluations which took as long as a month to occur. Earlier consultation with other specialists would have opened conversations for harm reduction efforts that could have allowed for alternatives to or delayed opioid deprescribing.

2) Intensified case management.

In cases of SUDs, comprehensive case management has been recognized as an important component of treatment. While heterogenous in makeup, case management can help improve engagement in SUD treatment and improve overall functioning.^12^ Notably, SUD treatment spans a care continuum, ranging from outpatient care to inpatient and residential treatment.^13^ Treatment resources matching the patient’s needs are not always going to be available through internal channels, and linkage to care can be cumbersome for a patient to navigate on their own. Case management includes ongoing assessments of patient needs and barriers with special attention to social determinates of health. Managers make and monitor referrals to internal and external resources and serve as an advocate for patients. They may help patients with Medicaid enrollment, connect them to financial and housing assistance resources, or facilitate appointment attendance through transportation vouchers or advocating for the batching of appointments. They may also serve as a liaison between team members, facilitating communication and collaboration.^12^

In our case, Mr. G had numerous psychosocial barriers, including low socioeconomic status, lack of health insurance, poor social support, and lack of transportation. Social work was contacted and assumed the primary role of case management in a more consultative and reactionary model. In retrospect, earlier proactive engagement in concert with other providers may have been more beneficial and allowed for the implementation of harm reduction efforts that better accommodated the patient’s unique social barriers.

3) Early connection to advanced non-opioid pain interventions.

Opioids have been the mainstay treatment of cancer-related pain. A history of SUD has been a factor steadily associated with increased risk for prescription opioid misuse.^14^ Based on a patient’s risk, a harm reduction strategy should be developed at the patient’s initial consultation. Described in more detail in dedicated publications, this may include use of buprenorphine as the first-line opioid analgesic, pill counts, urine drug tests, more frequent visits, overdose education and prevention efforts, etc.^1,7,8,11^ Pain is a multifaceted experience that involves physical, psychological, and spiritual aspects; thus, a multimodal approach is the most effective way to manage it in vulnerable populations. There should be an optimization of nonopioid analgesics, such as nonsteroidal anti-inflammatory drugs, antidepressant, anticonvulsants, and topical analgesic, based on the nature of the pain and concomitant symptoms. Nonpharmacologic therapies should also be explored, such as an early referral to an interventional pain specialist. Interventional techniques such as neuraxial analgesia, minimally invasive procedures for vertebral pain, sympathetic blocks for abdominal cancer pain, peripheral nerve blocks, and percutaneous cordotomy can help to provide localized pain relief and limit systemic opioid use.^15^ Acupuncture and massage have also been shown to reduce pain, as well as improve concurrent fatigue and insomnia symptoms.^16^

In the case of our patient, the delay in identifying his confounding SUD delayed the prioritization of targeted interventions. In addition, optimization of nonopioid interventions was severely limited by his difficultly attending appointments, likely due to his ongoing severe SUD and significant psychosocial barriers.

In summary, navigating the risks of prescribed opioids in high-risk cancer patients requires vigilance, multi-modal approaches, and close attention to the psychosocial context. While internal resources will vary widely depending on the practice setting, even resource-rich centers can fall victim to care fragmentation. Complicated cases may benefit from alternative modes of communication and collaboration deviating from the more typical patterns of referral, emphasizing earlier identification, collaboration, and proactive case management (Table 1).

**Table 1.** Care Delivery Considerations for High-Risk Opioid Patients

1) Reduce barriers to early specialist collaboration. A) Utilize resources for assessment and management across multiple disciplines. B) Develop forums for review independent of formal consultation. Examples include: i) Collaborative care model ii) Tumor board review 2) Explore pathways for escalated case management for patients with higher psychosocial needs or active substance use disorder. 3) Prioritize and troubleshoot barriers to early optimization of nonopioid medication and interventions.

## Abbreviations Used

OUD: opioid use disorder
PET: positron emission tomography
SUD: substance use disorder
